# Local versus bulk circular dichroism enhancement by achiral all-dielectric nanoresonators

**DOI:** 10.1515/nanoph-2022-0293

**Published:** 2022-08-12

**Authors:** Krzysztof M. Czajkowski, Tomasz J. Antosiewicz

**Affiliations:** Faculty of Physics, University of Warsaw, Pasteura 5, PL-02-093 Warsaw, Poland

**Keywords:** all-dielectric nanoresonators, circular dichroism, optical chirality, t-matrix method

## Abstract

Large optical chirality in the vicinity of achiral high-index dielectric nanostructures has been recently demonstrated as useful means of enhancing molecular circular dichroism. We theoretically study the spatial dependence of optical chirality enhancement in the vicinity of high-index dielectric nanodisks and highlight its importance for the design of nanophotonic platforms for circular dichroism enhancement. Using a T-matrix framework, we demonstrate that, depending on the disk aspect ratio, chirality is enhanced preferentially along different directions. We employ various statistical procedures, including surface, volume and orientation averaging, to predict enhancement of chiroptical effects and show that optimal properties of a nanostructure depend substantially on whether spatial maximum or average chirality enhancement is sought after. The results indicate that at times it is beneficial to sacrifice helicity preservation for a larger field enhancement. Similarly, the optimal choice of the nanostructure is influenced by presence of a substrate, which limits the space available to be occupied by analyte molecules and impacts the optical chirality in the vicinity of the nanostructure.

## 1 Introduction

Chirality is a feature of many biologically active molecules including drugs [1]. A molecule is chiral if its mirror image is non-superimposable on the molecule by any combination of rotations and translations. Enantiomers, two configurations of a chiral molecule, may exhibit drastically different biological activity. At an extreme, one enantiomer may have beneficial medicinal effects, while the other may be toxic [2]. Thus, determining molecular helicity is of paramount importance. An optical way to differentiate between enantiomers is circular dichroism (CD) spectroscopy, which relies on differential absorption of left- and right-handed circularly polarized light. Intrinsic molecular CD is low which leads to long acquisition times, decreased sensitivity and in turn diminishes the practicality of the method. At the same time, sensing of molecular chirality is of paramount importance e.g. in drug development. As shown by Tang and Cohen [3, 4], one approach to achieve the goal of substantial optical chirality enhancement (OCE) is using nanoantennas which locally enhance electromagnetic fields. This enhancement has been recently experimentally demonstrated to be useful for various types of optical spectroscopies utilizing chiroptical effects [5, 6]. Assuming that the molecule is a small object, which can be approximated by a non-radiating point dipole, its CD signal is proportional to the chiral part of its polarizability and optical chirality density.

The figure of merit in circular dichroism enhancement is the enhancement of chirality density $\left(f\left(\vec{r}\right)\right)$ , which is given by

(1) $$f\left(\vec{r}\right)=-Z\frac{Im\left({\vec{E}}^{*}\left(\vec{r}\right)\cdot \vec{H}\left(\vec{r}\right)\right)}{|{\vec{E}}_{0}{|}^{2}},$$

where *Z* is the relative impedance of the surrounding medium, $\vec{E}$ is the electric field, $\vec{H}$ is the magnetic field and ${\vec{E}}_{0}$ is the incident electric field amplitude. We assume here that the incident field is a left-handed polarized plane wave (hence the minus sign). Recently a set of design rules, which a suitable CD-enhancing nanostructure should fulfill, have been proposed [7]. The nanostructure should be achiral to avoid contamination of the CD signal of the molecule by the CD signal of the nanostructure itself. Otherwise, the CD signal could be generated by achiral molecules or the signal coming from the nanoparticle could be orders of magnitude larger than that of the molecule. Other rules include helicity preservation and a strong electromagnetic response. They can be readily elucidated by representing the electromagnetic field using helicity eigenstates ${\vec{G}}^{\pm }=\vec{E}\pm iZ\vec{H}$ [8, 9]. Then, helicity change can be defined as

(2) $$\Lambda \left(\vec{r}\right)=\frac{|{\vec{G}}^{-}\left(\vec{r}\right){|}^{2}}{|{\vec{G}}^{+}\left(\vec{r}\right){|}^{2}+|{\vec{G}}^{-}\left(\vec{r}\right){|}^{2}}$$

and the total intensity enhancement as

(3) $$G_{\text{enh}}\left(\vec{r}\right)=\frac{|{\vec{G}}^{+}\left(\vec{r}\right){|}^{2}+|{\vec{G}}^{-}\left(\vec{r}\right){|}^{2}}{|{\vec{G}}_{\text{inc}}^{+}\left(\vec{r}\right){|}^{2}},$$

where $G_{\text{inc}}^{+}$ corresponds to the incident field. Using helicity eigenstates, the enhancement of optical chirality density is given by

(4) $$f\left(\vec{r}\right)=\frac{|{\vec{G}}^{+}\left(\vec{r}\right){|}^{2}-|{\vec{G}}^{-}\left(\vec{r}\right){|}^{2}}{|{\vec{G}}_{\text{inc}}^{+}\left(\vec{r}\right){|}^{2}}=G_{\text{enh}}\left(\vec{r}\right)\left(1-\Lambda \left(\vec{r}\right)\right),$$

which highlights the design rules proposed by Graf et al. [7].

Since the pioneering works by Tang and Cohen [3, 4] and Graf et al. [7], several nanostructure-based concepts to enhance optical chirality density emerged. Initially, plasmonic nanostructures [10] have been used, including single gold nanoparticles [11], gold nanoparticle dimers [12], racemic nanoplasmonic arrays [13]. After the publication by Graf et al. [7], many nanostructures aiming at helicity preservation have emerged, such as Fabry–Perot cavities containing nanodisk arrays [6, 14], metal-dielectric nanoparticle dimers [15], 1D silicon nanoparticle arrays [16], silicon nanorods [17] and silicon nanodisks [18] to simultaneously attain helicity preservation and a strong electromagnetic response. A simpler route to helicity preservation is offered by isolated high-index dielectric (HID) nanoparticles such as silicon nanospheres [19–21] or nanodisks [22]. In the dipolar approximation minimization of helicity change requires fulfilling the Kerker conditions, namely equal electric and magnetic dipole scattering amplitudes [23]. Consequently, HID nanodisks with overlapping electric and magnetic resonances have been considered as optimal for optical chirality density enhancement.

At the same time, one has to note that the optical chirality density is a spatially dependent property, but CD is a macroscopic property determined by the integral of chirality density over the volume outside the particle

(5) $$C_{D}=\frac{\text{Re}\left(\alpha^{em}\right)\omega }{2c}\underset{V}{\int }f\left(\vec{r}\right)\;\text{d}V^{\prime },$$

where *V* is the volume occupied by the molecules and *α*^*em*^ is the chiral polarizability of the molecule. Chirality density averaged over a spherical surface has been proposed by Garcia-Etxarri and Dionne [20] as a close proxy of the bulk enhancement

(6) $$f_{\text{avg}}\left(r\right)=\frac{1}{4\pi }\overset{2\pi }{\underset{0}{\int }}\text{d}\phi \overset{\pi }{\underset{0}{\int }}f\left(\vec{r}\right)\text{sin}\theta \;\text{d}\theta .$$

Due to spatial dependence of $f\left(\vec{r}\right)$ , the attainable CD enhancement depends on the spatial distribution of the molecules and thus designing the nanostructure for CD enhancement might depend on the specific experimental scenario.

Here, we combine semi-analytical considerations based on T-matrix formalism and numerical modeling using the finite-difference time-domain (FDTD) method to find out effective means of enhancing optical chirality using an archetypical all-dielectric nanoresonator – namely, a dielectric nanodisk. We highlight and focus on a considerable difference between local and bulk/surface averaged enhancement of chirality and study the dependence of the optical chirality density enhancement for a nanodisk geometry for each type of spatial averaging or lack thereof. We show that the optimal nanodisk aspect ratio depends on the spatial extent in which the chiral molecules reside and that at times sacrificing helicity preservation for a stronger electromagnetic response in the volume occupied by the molecules is beneficial for optical chirality enhancement.

This work is structured as follows. First, we describe the theoretical approach used herein. Afterwards, we show that the direction along which chirality is enhanced and the maximal attainable chirality enhancement along each direction depend on the particle shape. We establish a simple semianalitical formalism based on the dipole approximation to rationalize the result. Next, we use the T-matrix method to find the surface averaged and orientation averaged chirality enhancement and combine these results with a helicity-preserving multipolar decomposition to find out the dependence of chirality enhancement on a particle’s aspect ratio and helicity change upon scattering. Finally, we use the FDTD method to corroborate the T-matrix derived local chirality enhancement maxima as well as averages of chirality enhancement over thin layers placed around the particle. We also study the impact of placing the particle on a substrate on anticipated chirality enhancement, because the presence of a substrate is a common factor in experimental platforms for nanophotonic enhancement of molecular circular dichroism.

## 2 Theory

As mentioned in the introduction, optical chirality density enhancement *f* is a useful figure of merit in circular dichroism. Here, we outline the theoretical framework to calculate chirality density using the scattering problem perspective and helicity multipoles [7]. Both ${\vec{G}}^{+}$ and ${\vec{G}}^{-}$ are separated into incident and scattered fields

(7) $${\vec{G}}^{\pm }={\vec{G}}_{\text{inc}}^{\pm }+{\vec{G}}_{\text{scat}}^{\pm },$$

where from now we omit the explicit dependence on $\vec{r}$ . Then the optical chirality enhancement can be split into interference (*f*_int_) and scattered parts (*f*_scat_) [24],

(8) $$f=1+f_{\text{int}}+f_{\text{scat}}$$

with

(9) $$f_{\text{int}}=\frac{2\text{Re}\left({\vec{G}}_{\text{scat}}^{+}\cdot {\vec{G}}_{\text{inc}}^{+}-{\vec{G}}_{\text{scat}}^{-}\cdot {\vec{G}}_{\text{inc}}^{-}\right)}{|{\vec{G}}_{\text{inc}}^{+}{|}^{2}}$$

and

(10) $$f_{\text{scat}}=\frac{1}{4}\frac{\left|{\vec{G}}_{\text{scat}}^{+}{|}^{2}-|{\vec{G}}_{\text{scat}}^{-}{|}^{2}\right|}{|{\vec{G}}_{\text{inc}}^{+}{|}^{2}}.$$

Note, that *f*_scat_ is strictly positive unless the structure scatters fields with right-handed helicity (−) more efficiently than those with left-handed (+) helicity. To the contrary, *f*_int_ has a plus or minus sign depending on the relative phase between the incident and scattered fields.

The scattered field is found either using the T-matrix approach or dipole approximation. The T-matrix has been recently shown as a valuable tool for analysis of chiral light–matter interactions [25]. In this work, the open source code SMUTHI is used to easily calculate the T-matrix and related quantities [26]. The T-matrix framework facilitates finding field distributions outside the circumscribing sphere of the particle by relying on field expansion using vector spherical wave functions (VSWFs) [27]. Due to the fact that we consider here chiral properties of optical fields, we use multipoles of pure helicity

(11) $$b_{l,m}^{\pm }=\frac{b_{l,m}^{e}\pm b_{l,m}^{m}}{\sqrt{2}}.$$

Using these multipole moments it is possible to calculate helicity change upon scattering simply as [7]

(12) $$\Lambda =\frac{\underset{l,m}{\sum }|b_{l,m}^{-}{|}^{2}}{\underset{l,m}{\sum }|b_{l,m}^{-}{|}^{2}+|b_{l,m}^{+}{|}^{2}}.$$

Another advantage of using VSWFs for field expansion is their orthogonality on a spherical surface [27] and hence the integral in Eq. (6) defining the surface averaged chirality enhancement can be evaluated analytically (see Supplementary Note S1 for details). We assume that the incident field is a left-handed circularly polarized plane wave with its wavevector aligned with the *z*-axis. We decompose the surface averaged OCE using the T-matrix approach (*f*^*T*^) by following Eq. (8) and find the interference $\left(f_{\text{int}}^{T}\right)$ and scattering $\left(f_{\text{scat}}^{T}\right)$ parts separately. The index *m* averages out due to the fact that we consider an axially symmetric particle which is illuminated by normally incident circularly polarized light. In this case, the interference part is

(13) $$f_{\text{int}}^{T}=\frac{1}{2}\underset{l}{\sum }\text{Im}\left(i^{l-1}w_{\text{ext},l}\left(k,r\right)b_{l}^{+}\right)$$

with

(14) $$\begin{array}{ll} w_{\text{ext},l}\left(k,r\right) & =j_{l}\left(kr\right)h_{l}\left(kr\right)\left(1+\frac{l\left(l+1\right)}{k^{2}r^{2}}\right)\\ & \;+\frac{1}{k^{2}r^{2}}\frac{\text{d}}{\text{d}kr}\left[krh_{l}\left(kr\right)\right]\cdot \frac{\text{d}}{\text{d}kr}\left[krj_{l}\left(kr\right)\right]. \end{array}$$

The scattering part is

(15) $$f_{\text{scat}}^{T}=\frac{1}{8}\underset{l}{\sum }w_{\text{scat},l}\left(k,r\right)\left(|b_{l}^{+}{|}^{2}-|b_{l}^{-}{|}^{2}\right)$$

with

(16) $$\begin{array}{ll} w_{\text{scat},l}\left(k,r\right) & =|h_{l}\left(kr\right){|}^{2}\left(1+\frac{l\left(l+1\right)}{k^{2}r^{2}}\right)+\frac{1}{k^{2}r^{2}}{\left|\frac{\text{d}}{\text{d}kr}\left[krh_{l}\left(kr\right)\right]\right|}^{2}. \end{array}$$

This result can be then further averaged over nanoparticle orientation using the concept of the orientation-averaged T-matrix (⟨*T*⟩) defined by Doicu et al. [27]. Average *f*_int_ is easily evaluated as

(17) $$〈f_{\text{int}}^{T}〉=2\underset{l}{\sum }\text{Im}\left(i^{l-1}w_{\text{ext},l}\left(k,r\right)〈b_{l}^{+}〉\right)$$

with $\left\langle \vec{b}\right\rangle$ defined as $\left\langle \vec{b}\right\rangle =\left\langle T\right\rangle \vec{a}$ , where $\vec{a}$ is the incident field coefficient vector. The scattered part is defined as

(18) $$〈f_{\text{scat}}^{T}〉={\vec{a}}^{†}〈W〉\vec{a}.$$

Matrix *W* is defined as *W* = *T*^†^*FT* with

$$F=\left[\begin{array}{cc} 0 & |h\left(kr\right){|}^{2}\\ \frac{1}{k^{2}r^{2}}{\left|\frac{\text{d}}{\text{d}kr}\left[krh_{l}\left(kr\right)\right]\right|}^{2}+l\left(l+1\right){\left|\frac{h\left(kr\right)}{kr}\right|}^{2} & 0 \end{array}\right].$$

Its orientation average is given by

(19) $$\left\langle {\left(W\right)}_{l,m,l^{\prime },m^{\prime }}^{i,j}\right\rangle =\delta_{mm^{\prime }}\delta_{ll^{\prime }}{\hat{t}}_{l}^{ij}$$

with

(20) $${\hat{t}}_{l}^{ij}=\frac{1}{2l+1}\underset{m^{\prime }}{\sum }{\left(W\right)}_{l,m,l^{\prime },m^{\prime }}^{ij}$$

Here, *i* and *j* index the dipole types (mag and el). The details of the orientation averaging procedure for *f*^*T*^ are given in Supplementary Note S2.

As shown in the previous paragraphs, the spherical basis is useful for expressing surface and orientation averages of optical chirality enhancement. Directional OCEs are more readily evaluated in the Cartesian basis. Here, we resort to the dipole approximation as it allows for relatively compact expressions that describe the OCE along specified directions and its value when averaged over a spherical surface. Instead of the T-matrix, polarizability is used in the dipolar approximation to describe the nanoparticle properties. The relationship between electric/magnetic polarizability (in CGS units) and the corresponding T-matrix element is

(21) $$\alpha^{e/m}=-i\frac{3}{2k^{3}}T^{ee/mm}.$$

The polarizability is then transformed into multipoles with defined helicity, where $\alpha^{\pm }=\left(\alpha^{e}\pm \alpha^{m}\right)/\sqrt{2}$ . The enhancement along *x* and *y* directions can be calculated as

(22) $$\begin{array}{ll} f_{xy} & =1+\left(|\alpha^{+}{|}^{2}-|\alpha^{-}{|}^{2}\right)w_{xy}\\ & \;+\text{Re}\left(\alpha^{+}\frac{\text{exp}\left(\text{i}kr\right)\left(k^{2}r^{2}+1-ikr\right)}{\sqrt{2}r^{3}}\right), \end{array}$$

with *w*_*xy*_ = (2*k*^4^*r*^4^ + 4*k*^2^*r*^2^ + 5)/(4*r*^6^). The enhancement along *z* is

(23) $$\begin{array}{ll} f_{z_{-}} & =1+\left(|\alpha^{+}{|}^{2}-|\alpha^{-}{|}^{2}\right)\frac{1}{2r^{6}}+|\alpha^{+}{|}^{2}\frac{4k^{4}r^{4}}{2r^{6}}\\ & \;+\text{Re}\left(\alpha^{+}\frac{\sqrt{2}\left(2k^{2}r^{2}-1\right)+i2\sqrt{2}kr}{r^{3}}\right) \end{array}$$

below the particle and

(24) $$\begin{array}{ll} f_{z_{+}} & =1+\left(|\alpha^{+}{|}^{2}-|\alpha^{-}{|}^{2}\right)\frac{1}{2r^{6}}+|\alpha^{-}{|}^{2}\frac{4k^{4}r^{4}}{2r^{6}}\\ & \;+\frac{\sqrt{2}}{r^{3}}\text{Re}\left(\alpha^{+}\exp\left(2\text{i}kr\right)\right) \end{array}$$

above the particle. The dipolar approximation can be also used to perform surface averaging, giving

(25) $$\begin{array}{ll} f_{\text{avg}} & =1+w_{\text{avg}}\left(k,r\right)\left(\left(|\alpha^{+}{|}^{2}-|\alpha^{-}{|}^{2}\right)-\frac{3\text{Im}\left(\alpha^{+}\right)}{\sqrt{2}k^{3}}\right)\\ & \;+\text{Re}\left(\alpha^{+}\frac{\text{exp}\left(2\text{i}kr\right)\left(-6kr+i\left(3-4k^{2}r^{2}\right)\right)}{\sqrt{2}k^{3}r^{6}}\right) \end{array}$$

with $w_{\text{avg}}\left(k,r\right)=\left(2k^{4}r^{4}+2k^{2}r^{2}+3\right)/\left(3r^{6}\right)$ . Note, that this extends other dipolar approximations present in the literature [20, 28, 29] by not resorting to the quasi-static approximation and considering OCE along specific directions and its surface average.

## 3 Results and discussion

**Figure 1:**
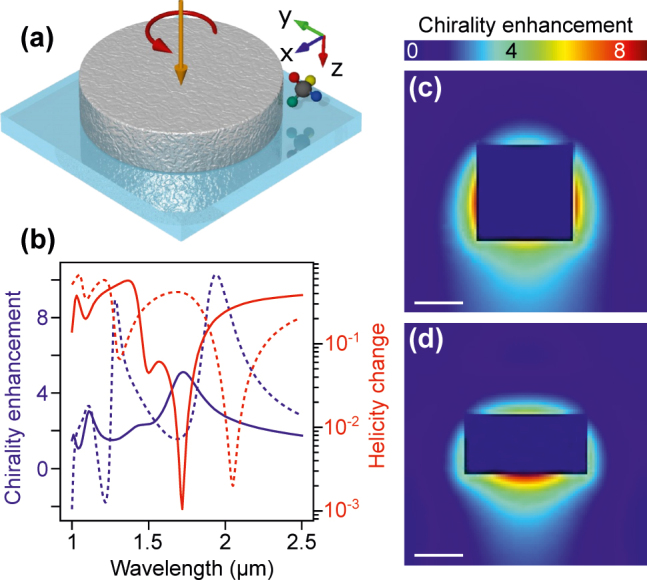
Calculation of optical chirality enhancement for a model HID nanodisk. (a) Schematic representation of the studied system. High index (*n* = 4 to mimic silicon) dielectric nanodisk is used to enhance the optical chirality density at molecule location. The system is illuminated by a left-handed circularly polarized light and embedded in water (*n* = 1.33). (b) Comparison of helicity change and chirality enhancement spectra for the two disks with varying AR. Solid lines: A=0.5 , dashed lines: A=1 . Chirality enhancement is averaged over a spherical surface with radius 50 nm larger than that of a disk. For the disk with A=0.5 maximal enhancement is matched with helicity change minimum, while for A=1 the two are significantly shifted with respect to each other. Maximum surface averaged enhancement for the disk with A=1 is larger than for the disk with A=0.5 , yet it corresponds to large helicity change on the order of 10%. Spatial maps of chirality enhancements for nanodisks with ARs (c) A=1 and (d) A=0.5 (the scale bars are 200 nm long). Note how, A=0.5 leads to enhancement predominantly along the *z*-axis below the particle, while when A=1 chirality is enhanced preferentially around the middle (circumference).

Dielectric nanodisks provide local enhancement of optical chirality, which in turn results in enhancement of circular dichroism of molecules localized in the vicinity of such nanodisks. Figure 1 presents maps of local enhancement of chirality for two exemplary HID nanodisks with equal volume of *V* = 5 × 10^7^ nm^3^ and different aspect ratio (AR) defined as A=H/D with *H* being the thickness and *D* the diameter of the nanodisk. The illumination is a plane wave propagating in the −*z*-direction with right-handed circular polarization. Its wavelength is tuned for each disk to obtain maximal local chirality enhancement. The maximum attainable local enhancement of up to 9 is provided by a nanodisk with AR of about 0.5. The optical response of this nanodisk consists of overlapping electric and magnetic dipole resonances, thereby fulfilling the Kerker condition while maintaining a strong resonant electromagnetic response. Such disks have been considered optimal for chirality enhancement. At the same time, we observe that when AR is changed so that the disk thickness is equal to its diameter, the maximal local enhancement does not change substantially (see Figure 1(b)). Instead, other changes are more noticeable. Namely, the locations at which chirality enhancement is maximized are very different for each of the two analyzed cases. For the disk with A=0.5 , Figure 1(d), the maximum is located below the particle in the *z*-direction, which coincides with the propagation direction of the incident wave. In contrast, for the disk with A=1 , Figure 1(c), the maximum is located at the sides of the disk around its circumference. Enhancement at the top and bottom surfaces of this disk is substantially lower (around 5).

To quantify chirality enhancement without having to specify the location, we use *f*_avg_. The average chirality enhancement spectra for both nanodisks under consideration are presented in Figure 1(b) along with corresponding helicity change spectra calculated using the T-matrix method. The size of the spherical shell is set so that the distance between particle surface and the shell surface is 50 nm. Despite the fact that the maximal attainable local enhancement is provided by nanodisk with A=0.5 , it is the other nanodisk that enables higher average chirality enhancement maximum.

**Figure 2:**
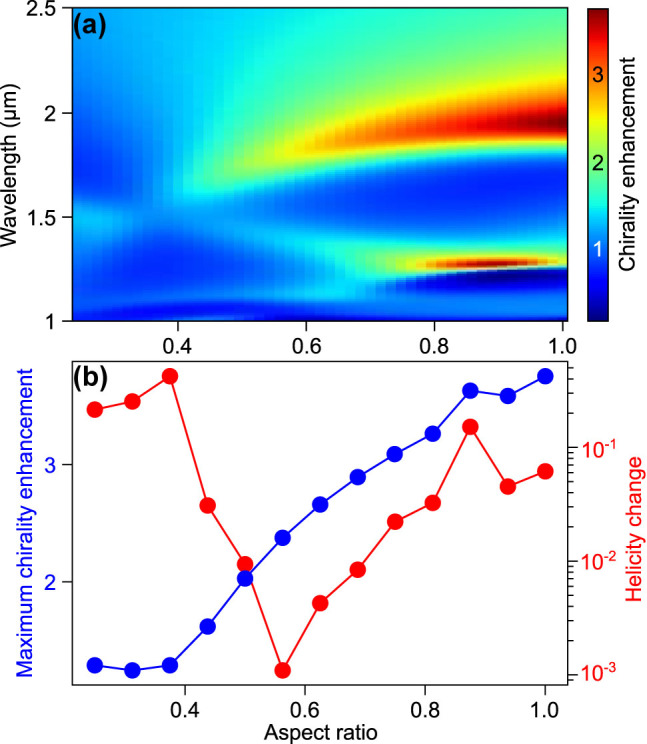
Tuning chirality enhancement with aspect ratio. (a) Surface averaged chirality enhancement spectra as a function of aspect ratio A for water-embedded HID nanodisks with fixed volume of 5 × 10^7^ nm^3^. For flat disks the enhancement is generally low. For tall disks two contributions are present: dipolar around 1750–2000 nm and quadrupolar around 1250 nm. (b) Maximum surface averaged chirality enhancement as a function of AR and corresponding helicity change. Note how chirality enhancement is monotonic with aspect ratio (except near A=0.9 , where the quadrupole mode leads to maximum enhancement), while it is not correlated with the corresponding helicity change.

Next, we investigate how chirality enhancement and helicity change based on the far-field calculation vary over a broad range of nanodisk aspect ratio. To that end, we utilize the T-matrix formalism which facilitates rapid calculation of chiral properties of the electromagnetic field scattered by nanoresonators as shown in the Theory section. The surface averaged chirality enhancement spectrum as a function of nanodisk aspect ratio is presented in Figure 2(a). For flat disks with AR below 0.4, the observed enhancement is almost negligible. Above this value, we observe that with increasing AR the surface averaged chirality enhancement increases. Furthermore, for nanodisks with sufficiently large AR two maxima in the chirality enhancement spectrum are present. The one corresponding to the larger wavelength results from an interplay of electric and magnetic dipoles, while the second one corresponds to quadrupoles. The quadrupolar peak is narrower than the dipolar one, which is typical for optical cross-section spectra of HID nanodisks. Depending on AR, the quadrupolar peak might have a larger amplitude than the dipolar one, but it is worth noting that the considered structures lack absorption (having used *n* = 4 only), which tends to diminish the impact of quadrupoles on the optical properties (although for Si this would happen only for significantly shorter wavelengths).

In Figure 2(b), we compare the maximal chirality enhancement (maximum is taken over wavelength, while averaging is performed over the particle surface) with the helicity change calculated based on helicity multipole decomposition (see Eq. (12)) at the wavelength corresponding to the chirality enhancement maximum. The helicity change features a very clear minimum at AR of approximately 0.55, where its value is about 0.1%. This due to the fact that the disk with this AR fulfills the Kerker condition, which is known to be the condition for minimal helicity change [23]. An increasing value of helicity change is observed as AR shifts away from the value corresponding to the Kerker condition. Over 10% of helicity change is observed for very small aspect ratios and for AR of ca. 0.9. While no clear correlation between surface averaged chirality enhancement and helicity change is observed, elongated cylinders perform better than flat disks and not even a local maximum is observed for the AR corresponding to the helicity change minimum. Surface average chirality enhancement almost exclusively increases with increasing AR. The exception is a local maximum around AR of 0.9, which stems from a narrow quadrupole peak. This peak gives the maximum chirality enhancement while simultaneously yielding an exceptionally high helicity change.

**Figure 3:**
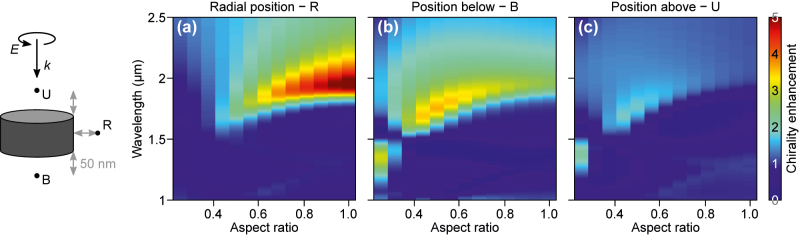
Chirality enhancement spectra for high-index nanodisks calculated along specific directions: (a) in the radial direction, (b) in *z* below the disk and (c) in *z* above the disk at a distance of 50 nm away from each particle. The positions are marked in the cartoon on the left. Here, semi-analytical expressions obtained in the dipole approximation are used. Therefore, quadrupole maxima are not present. Note how maxima of chirality enhancements appear for different aspect ratio for each direction. Also, maximal attainable value for the *z* direction is smaller than that in the radial direction.

To gain further physical insight, we resort to the dipolar approximation, which enables calculation of optical chirality enhancement along the *x*- and *z*-axis via Eqs. (22) and (23), respectively (the *x* direction is equivalent to a radial one due to axial symmetry). Figure 3 presents the dependence of the chirality enhancement spectrum on the nanodisk aspect ratio in the radial direction and along the propagation direction *z* below and above the disk. We fix the distance between the nanodisk surface and the observation point (molecule) at 50 nm. The result obtained for OCE calculated along the radial direction is qualitatively similar to the surface averaged chirality enhancement obtained using the T-matrix approach (shown in Figure 2), with the exception of the quadrupolar feature at 1300 nm, which is omitted in this qualitative description. Despite this simplification, we observe a reasonably good agreement between the dipole approximation calculations and FDTD simulation results as shown in the Supporting Material Figure S1.

The results obtained for points along the radial and longitudinal/*z* directions are qualitatively very different (cf. Figure 3). The chirality enhancement along the radial direction is maximized by nanodisks with large aspect ratios following a similar dependence to that for the surface averaged chirality. In contrast, the enhancement along *z* is maximized by choosing an aspect ratio around 0.5, which corresponds to fulfilling the Kerker condition. With increasing aspect ratio, the chirality enhancement decreases while maintaining a similar wavelength dependence to those observed for surface averaged chirality. These observations are in agreement with the FDTD calculations presented in Figure 1. Both methods lead to the conclusion that for small aspect ratios the enhancement is predominantly in *z*, while for larger aspect ratios, it is observed predominantly in the radial direction. The enhancements observed along *z* are smaller than that in the radial direction in dipolar calculations, while the converse is true for FDTD calculations. We attribute this difference to the fact that when the dipole approximation is used the fields along *z* are calculated above the particle, while the enhancement is more prominent below the particle.

**Figure 4:**
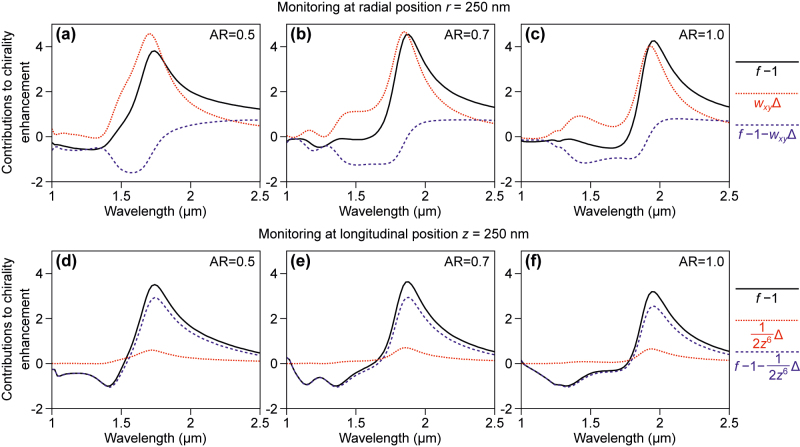
Various contributions to chirality enhancement (*f*) spectra in dipolar approximation: chirality enhancement minus one, term proportional to Δ = |*α*^+^|^2^ − |*α*^−^|^2^ and the remaining part that depends on *α*^+^ (independent on *α*^−^). Selected values of aspect ratio are being used. Top: at a single point with *r* = 250 nm, at a single point with *z* = 250 nm.

As readily seen from Eqs. (22) and (23), placing the sensed entity along different axes has a significant impact on the individual terms to optical chirality enhancement, which depends on the wavenumber and nanoparticle-molecule center-to-center distance. Here, we fix the displacement of the molecule along the radial direction or *z* at 250 nm and present the wavelength dependence of OCE on two relevant terms: the one proportional to Δ(≡|*α*^+^|^2^ − |*α*^−^|^2^) and the remaining term. These data are plotted in Figure 4 for selected aspect ratios of the nanodisk. Observation of large OCE requires simultaneously a large Δ-proportional term and interference (remaining) term. As seen in the top panel of the figure, this is not true for the Kerker disk, which offers the largest Δ proportional term and a large negative interference term, which diminishes substantially the observed enhancement. With the position of the molecule being fixed (instead of the particle surface-molecule distance), the OCE is almost independent of the aspect ratio as with increasing aspect ratio the Δ proportional term decreases, but the interference term increases to compensate. Eventually, for aspect ratio of 1, the interference term is positive.

The decomposition of optical chirality enhancement along *z* reveals very different trends than in the *x*-axis case. The Δ-proportional term decays with the center-to-center distance as *r*^−6^, which means that it contributes very little to the actual result. This is clearly seen in the bottom panel of Figure 4. This is a rather striking observation. It indicates that *α*^+^ rather than Δ determines the magnitude of OCE. Consequently, OCE does not require as strict a helicity preservation as it would be if the Δ-proportional term would be dominant. It also means that the overlap of electric and magnetic resonances is useful for obtaining large *α*^+^ rather than producing a large contrast between *α*^+^ and *α*^−^ in this case. It can be equally well replaced by a particle that simply features a strong *α*^+^ response. As an example, plasmonic particles that do not feature a magnetic dipole response would have *α*^+^ = *α*^−^ = *α*_*e*_, which would lead to equal amounts of + and − helicity being produced by scattering of light with + helicity. Hence, Δ would equal to zero. At the same time, *α*^+^ can be large and lead to substantial optical chirality enhancement.

**Figure 5:**
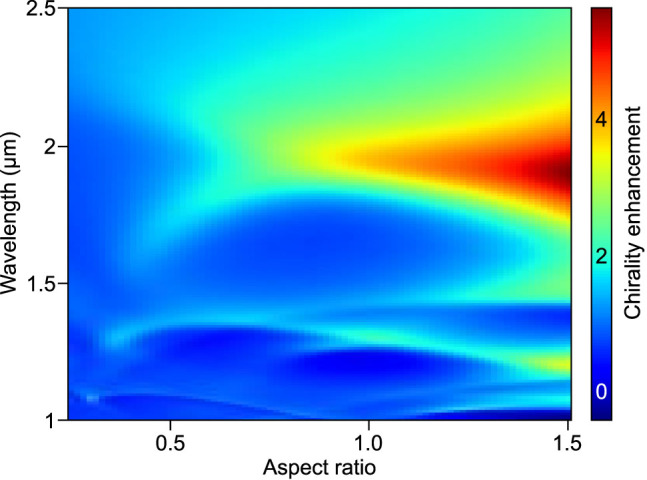
Orientation averaged chirality enhancement as a function of A obtained using the T-matrix method. In this scenario, chirality is enhanced predominantly by dipole modes with maximal enhancements observed for particles with large aspect ratios.

The strength of the T-matrix method lies also in its ability to yield optical quantities averaged over nanoparticle orientations [30]. Orientation averaging is especially useful for predicting the properties of nanoparticles in solutions, in which nanoparticles are of arbitrary orientation and thus orientation averaging is necessary for this task. Here, we utilize the tools developed in the Theory section to (see Eqs. (17) and (18)) to find the dependence of orientation averaged chirality enhancement on the nanodisk aspect ratio. The results of this study are presented in Figure 5. The size of the spherical surface over which spatial averaging is performed is determined such that the distance between the nanodisk surface and the sphere along *x* is 50 nm. We observe that the orientation averaged OCE follows similar trends as surface averaged chirality presented in Figure 2. At the same time, while surface averaged chirality featured a prominent quadrupolar feature for large aspect ratio, this feature is much less intense when averaged over particle orientation.

The main limitation of the T-matrix method (also in the dipolar approximation) is that it does not yield reliable values of the near fields located within the smallest circumscribing sphere of a nanoparticle (see Figure S2 for comparison of OCE between FDTD and the T-matrix method). Thus, it is not possible to perform a volume integral to calculate volumetric circular dichroism enhancement. Also, the method of surface averaging proposed here does not extend easily to particles on a substrate as it would require accounting for substrate-mediated self-coupling. To circumvent these issues, we complement the T-matrix method by FDTD simulations, which are substantially more resource- and time-consuming, but are reliable enough to yield accurate near fields and particles placed on a substrate with a refractive index of *n*_sub_.

**Figure 6:**
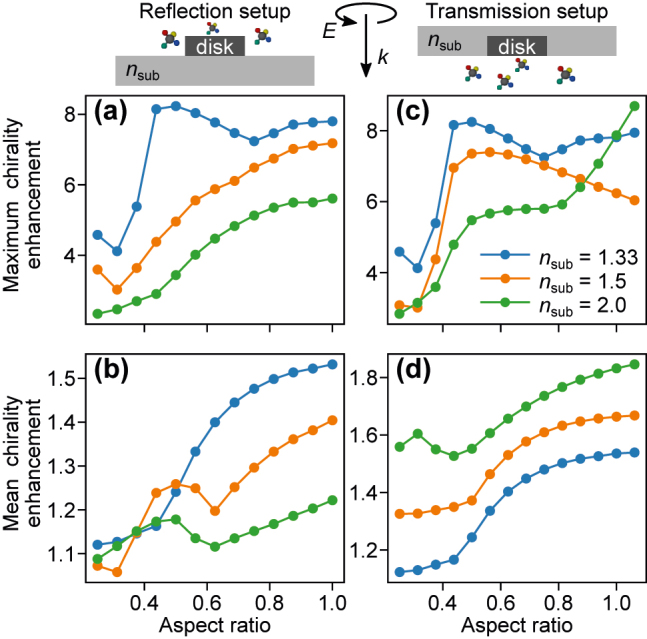
Chirality enhancement of a particle on a substrate. (a, c) Maximum and (b, d) mean chirality enhancement as a function of AR for varying substrate refractive index: index-matched (1.33), glass (1.5) and high-index dielectric (2) in (a, b) a reflection and (c, d) transmission configuration. Here, the statistics are taken over the upper half-space of the simulation, with the exception of index-matched case in which the both half-spaces are used (assuming that in a solution the specimen can take arbitrary position, while in the presence of a substrate, molecules cannot enter the substrate). The space occupied by the particle is always excluded from the analysis.

We use a total-field/scattered-field source and monitor both the electric and magnetic fields inside a box with a side length of 500 nm, which is large enough to fit all disks with various ARs without changing its size. As a figure of merit of bulk CD enhancement, we use the chirality enhancement averaged over the box volume excluding the particle volume and the substrate. In a homogeneous medium, the integration extends also over the bottom half-space. We compare such a volumetric mean of OCE to its spatial maximum over the integration volume in Figure 6 for two configurations in reflection (with the electric field incident onto the substrate) and transmission (with the field incident from the substrate). Clearly, the maximum attainable local enhancement varies greatly from the volume average, while both also depend on the illumination conditions.

The results in Figure 6 for *n*_sub_ = 1.33 confirm the validity of the T-matrix method based analysis presented above. Local OCE in a homogeneous environment is maximized by a Kerker disk as shown by other authors earlier [7, 22]. This local maximum is found below the particle as indicated in Figure 1(a). Nanodisks with larger aspect ratios perform worse than a corresponding Kerker disk in terms of the maximal local enhancement. However, the difference is not large – it is around 8 for the Kerker disk and for the disk with its aspect ratio equal to 1. The situation changes when the nanodisk is placed on a substrate. Then, the results depend on the choice of the setup. In the reflection setup, we assume that the molecules cannot reside in the bottom half-space, where the maximum for the Kerker disk is localized. In this case, utilizing enhancement along *x*-axis (radial displacement) is more beneficial, which shows nanodisks with large aspect ratios to be better at enhancing chirality than the Kerker disk. We observe that the presence of a substrate is detrimental to chirality enhancement with maximal attainable enhancement (for nanodisk aspect ratio of 1) of about 7 for a glass (*n*_sub_ = 1.5) substrate and about 5 for an index (*n*_sub_ = 2) substrate.

The volume averaging results obtained for the reflection setup with FDTD are presented in Figure 6(b). When the nanodisk is embedded in a homogeneous environment, the T-matrix results are qualitatively reproduced. The volume averaged OCE increases with increasing nanodisk aspect ratio. However, it reaches only a very modest value of about 1.5 for the largest AR – a value far smaller than of the local maximum. This indicates that nanostructures are most promising for enhancing chirality in their close vicinity, but not for enhancing the chiroptical response in bulk. The enhancement is additionally limited if the substrate is present. Especially, in this case the dependence on aspect ratio is no longer monotonic and exhibits a minimum for AR of approximately 0.6. As shown in Figure S3, the detrimental effect of the substrate in the reflection setup stems from the fact that with increasing *n*_sub_ OCE is increasingly directed towards the bottom half-space, which cannot be occupied by molecules in this configuration.

Both local and volume averaged OCE results are vastly different if the transmission setup is used as shown in Figure 6(c) and (d). When a glass substrate is used, the local OCE is maximized by a Kerker disk and equal to about 7, while disks with larger AR enable a lower OCE of about 6. However, if a large refractive index substrate is used, the local maximum of the OCE does not correspond to the Kerker disk. Instead, the high-aspect ratio disk outperforms it and leads to OCE of over 8, which is larger than that observed in a homogeneous medium. To rationalize this result, we present the spatial OCE maps in Figure S4. In the transmission setup the disk is embedded in the substrate and because light is incident from the substrate, molecules are placed in the area of high OCE below the nanodisk. At the same time, the impact of increasing *n*_sub_ depends on the nanodisk aspect ratio. For the Kerker disk, the OCE enhancement is large below the disk if the refractive index of the substrate is matched to that of the water and decreases with increasing *n*_sub_. The contrary is true for the nanodisk with AR of 1. Then, the OCE in a homogeneous medium is enhanced predominantly in the substrate and is redirected towards the bottom half-space (and thus the molecules) as *n*_sub_ increases. This explains the observed trends in Figure 6(c). Due to this increasing directionality of the OCE towards the bottom half-space, the volume averaged OCE increases with increasing *n*_sub_ as shown in Figure 6(d). Consequently, in this configuration we observe larger average values up to over 1.8 for AR of 1.0 and high-index dielectric substrate.

**Figure 7:**
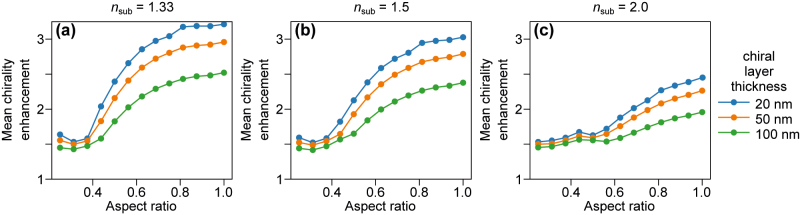
Mean chirality enhancement in thin layers (thickness from 20 to 100 nm) surrounding the nanodisk using the reflection configuration. (a) Disk embedded in water *n*_sub_ = *n*_env_, (b) glass with *n* = 1.5, and (c) high-index dielectric *n* = 2. It is clear that a substrate is detrimental to the device performance, while the dependence on AR is always close to that obtained by surface averaging.

As a last step of this study, we analyze enhancement of optical chirality in a thin layer surrounding the nanodisk to mimic a monolayer adsorption experiment in reflection configuration. This case resembles the one of surface averaged chirality, but here we adopt the shape of the nanoparticle when averaging. The results are presented in Figure 7. We observe that the layer averaged values follow the volumetric enhancement trends presented in Figure 6. Because optical chirality decays with an increasing distance from the nanodisk, thin layers result in larger mean enhancement compared to volumetric enhancement. Also, OCE decreases with increasing layer thickness. As in the other cases, the presence of the substrate limits the attainable enhancement.

## 4 Conclusions

In summary, we show that due to optical chirality being a local property with considerable spatial variation, there is a significant difference in the observed circular dichroism depending on the distribution of the analyte molecules. In terms of modelling, this implies that different statistical procedures are necessary to predict an experiment’s outcome depending on how it is designed. We have compared three possible scenarios: local enhancement, surface/layer enhancement and volumetric enhancement of optical chirality using achiral high-index dielectric nanocylinders. While such nanoresonators have been widely investigated in terms of their local capabilities to enhance chirality, a more comprehensive study was lacking. Here, we demonstrate that if average enhancement is sought, the optimal properties of a nanostructure to enhance chirality are considerably different that those optimizing the spatial chirality maximum. Even when local chirality enhancement is considered, the optimal nanostructure geometry depends on the point in space in which one wants to enhance chirality and on the choice of substrate. Our approach shows that this is the consequence of the fact that both field enhancement and helicity preservation must be maximized to observe high enhancement. However, obtaining both conditions simultaneously may not be possible. Hence, nanostructures which preserve helicity may be less suitable than those that do not provide perfect helicity preservation, when the former do not feature a strong electromagnetic response. It is worth noting that this is not in tension with the results presented in Ref. [7] that assumed a fixed field enhancement. Instead, our work extends those results by focusing on the case when the field enhancement is not fixed. We also show that the role of the substrate may be positive or detrimental depending on the particle shape and the experimental configuration. We anticipate that this work provides a useful theoretical framework for studying spatial dependence of optical chirality and that it facilitates designing of future experimental realizations of nanophotonic platforms for chiral molecule sensing.

## Supplementary Material

Supplementary Material Details
