# Supplementary material for: Local versus bulk circular dichroism enhancement by achiral all-dielectric nanoresonators
Source: Nanophotonics. 2022 Aug 12;11(18):4287–97. doi: 10.1515/nanoph-2022-0293 (PMC11501269; doi:10.1515/nanoph-2022-0293)
Supplement: Supplementary file 1 — Supplementary Material Details [file j_nanoph-2022-0293_suppl.pdf]

Supplementary Material

Local versus bulk circular dichroism enhancement by achiral all-dielectric nanoresonators

Krzysztof M. Czajkowski and Tomasz J. Antosiewicz

Contents

Supplementary Notes **2**
S1. Chirality enhancement in dipolar approximation 2
S2. Bulk chiral sensing in T-matrix formalism 2
T-matrix formalism 2
Spatial averaging of the optical chirality enhancement 3
Orientation averaging of surface averaged chirality enhancement 4

Supplementary Figures **6**
S1. Accuracy of OCE from FDTD and T-matrix 6
S2. Impact of multipole truncation order on OCE in T-matrix 6
S3. Optical chirality enhancement maps in reflection 7
S4. Optical chirality enhancement maps in transmission 8

Supplementary Notes

Supplementary Note S1: Chirality enhancement in dipolar approximation.

To calculate the chirality enhancement for scatterers in a homogeneous environment in the dipolar approximation the field is decomposed into incident and scattered components

$$\vec{E} = \vec{E}_{inc} + \vec{E}_{scat}, \tag{S1}$$

$$\vec{H} = \vec{H}_{inc} + \vec{H}_{scat}. \tag{S2}$$

The scattered field is calculated using the Green tensor [1]. We utilize CGS units for this Appendix.

$$\vec{E}_{scat} = G\vec{p} - (\vec{g} \times \vec{m}), \tag{S3}$$

$$\vec{H}_{scat} = G\vec{m} + (\vec{g} \times \vec{p}), \tag{S4}$$

with

$$G = \frac{e^{ikr}}{r}\left(\left(k^2 + \frac{ik}{r} - \frac{1}{r^2}\right)\hat{I} + \left(-k^2 - \frac{3ik}{r} + \frac{3}{r^2}\right)\hat{r}\hat{r}\right) \tag{S5}$$

and

$$\vec{g} = \frac{ie^{ikr}k(ikr-1)}{r^2}\hat{r}. \tag{S6}$$

Dipole moments ($\vec{p}$ and $\vec{m}$) of an achiral structure are related to the incident fields via polarizability tensors (α^e and α^m). Assuming that the field propagates in the z-direction and that the nanostructure enhancing chirality is axially symmetric and achiral,

$$\vec{p} = \alpha^{el}_{xx}\vec{E}_{inc}, \tag{S7}$$

$$\vec{m} = \alpha^{mag}_{xx}\vec{H}_{inc}. \tag{S8}$$

The optical chirality density enhancement is found by evaluating G and $\vec{g}$ along x direction (with spherical coordinates $\theta = \frac{\pi}{2}, \phi = 0$) and along z direction (with spherical coordinates $\theta = \phi = 0$). Then, the resulting $\vec{E}$ and $\vec{H}$ are inserted in eq. 1 in the main text. In order to convert the results to helicity preserving dipoles, we utilize the relation presented in the main text,

$$\alpha^{\pm} = \frac{\alpha^{el} \pm \alpha^{mag}}{\sqrt{2}}. \tag{S9}$$

Supplementary Note S2: Bulk chiral sensing in T-matrix formalism.

T-matrix formalism

In the T-matrix formalism scattered electric and magnetic fields are expanded into radiating VSWFs as

$$\vec{E}_{scat}(\vec{r}) = \sum_{l,m} b^{mag}_{l,m}\vec{M}^3_{l,m}(\vec{r}) + b^{el}_{l,m}\vec{N}^3_{l,m}(\vec{r}) \tag{S10}$$

and

$$\vec{H}_{scat}(\vec{r}) = \frac{1}{iZ}\sum_{l,m} b^{el}_{l,m}\vec{M}^{1,3}_{l,m}(\vec{r}) + b^{mag}_{l,m}\vec{N}^{1,3}_{l,m}(\vec{r}), \tag{S11}$$

while incident electric and magnetic fields are expanded into regular VSWFs

$$\vec{E}_{inc}(\vec{r}) = \sum_{l,m} a^{mag}_{l,m}\vec{M}^1_{l,m}(\vec{r}) + a^{el}_{l,m}\vec{N}^1_{l,m}(\vec{r}) \tag{S12}$$

and

$$\vec{H}_{inc}(\vec{r}) = \frac{1}{iZ}\sum_{l,m} a^{el}_{l,m}\vec{M}^1_{l,m}(\vec{r}) + a^{mag}_{l,m}\vec{N}^1_{l,m}(\vec{r}) \tag{S13}$$

with $\vec{M}_{l,m}(\theta,\varphi)$ and $\vec{N}_{l,m}(\theta,\varphi)$ being VSWFs. VSWFs are defined as

$$\vec{M}^{1,3}_{l,m}(k\vec{r}) = z^{1,3}_n(kr)\vec{m}_{l,m}(\theta,\varphi), \tag{S14a}$$

$$\vec{N}^{1,3}_{l,m}(k\vec{r}) = \sqrt{\frac{n(n+1)}{2}}\frac{z^{1,3}_n(kr)}{kr}Y_{l,m}(\theta,\varphi)\vec{e}_r + \frac{\frac{\mathrm{d}}{\mathrm{d}kr}[krz^{1,3}_n(kr)]}{kr}\vec{n}_{l,m}(\theta,\varphi), \tag{S14b}$$

with $\vec{m}_{l,m}(\theta,\varphi)$ and $\vec{n}_{l,m}(\theta,\varphi)$ being VSH, $Y_{l,m}$ being scalar spherical harmonics as defined by Doicu et al. [2]. $z_n^{1,3}$ is spherical Bessel functions (j_n) for VSWF of the regular type (upper index equal to 1) and spherical Hankel functions (H_n) for VSWF of the radiating type (upper index equal to 3).

The incident field expansion coefficients ($a_{l,m}^{mag}, a_{l,m}^{el}$) are related to the scattered field expansion coefficients via T-matrix (T)

$$\begin{bmatrix} b^{mag} \\ b^{el} \end{bmatrix} = \begin{bmatrix} T^{mm} & T^{me} \\ T^{em} & T^{ee} \end{bmatrix} \begin{bmatrix} a^{mag} \\ a^{el} \end{bmatrix}. \tag{S15}$$

The following orthogonality relations apply to VSHs

$$\int_0^{2\pi}\int_0^{\pi} \vec{m}_{l,m}(\theta,\varphi)\cdot\vec{m}^*_{l',m'}(\theta,\varphi)\sin\theta \mathrm{d}\theta\mathrm{d}\varphi = = \int_0^{2\pi}\int_0^{\pi} \vec{n}_{l,m}(\theta,\varphi)\cdot\vec{n}^*_{l',m'}(\theta,\varphi)\sin\theta \mathrm{d}\theta\mathrm{d}\varphi = \pi\delta_{mm'}\delta_{ll'} \tag{S16}$$

and

$$\int_0^{2\pi}\int_0^{\pi} \vec{m}_{l,m}(\theta,\varphi)\cdot\vec{n}^*_{l',m'}(\theta,\varphi)\sin\theta \mathrm{d}\theta\mathrm{d}\varphi = 0. \tag{S17}$$

For the scalar spherical harmonics a similar orthogonality relation is

$$\int_0^{2\pi}\int_0^{\pi} Y_{mn}(\theta,\varphi)Y_{m'n'}(\theta,\varphi)\sin\theta \mathrm{d}\theta\mathrm{d}\varphi = 2\pi\delta_{m,-m'}\delta_{nn'}. \tag{S18}$$

Using these relations one can show that

$$\int_0^{2\pi}\int_0^{\pi} \vec{M}^3_{l,m}(\theta,\varphi)\cdot\vec{M}^{3*}_{l,m}(\theta,\varphi)\sin\theta \mathrm{d}\theta\mathrm{d}\varphi = \pi\left(\frac{1}{k^2r^2}|\frac{\mathrm{d}}{\mathrm{d}kr}[krh(kr)]|^2 + l(l+1)\left|\frac{h(kr)}{kr}\right|^2\right), \tag{S19}$$

$$\int_0^{2\pi}\int_0^{\pi} \vec{N}^3_{l,m}(k,\vec{r})\cdot\vec{N}^{3*}_{l,m}(k,\vec{r})\sin\theta \mathrm{d}\theta\mathrm{d}\varphi = \pi|h(kr)|^2, \tag{S20}$$

$$\int_0^{2\pi}\int_0^{\pi} \vec{M}^3_{l,m}(k,\vec{r})\cdot\vec{M}^{1*}_{l,m}(k,\vec{r})\sin\theta \mathrm{d}\theta\mathrm{d}\varphi = = \pi\left(\frac{1}{k^2r^2}\frac{\mathrm{d}}{\mathrm{d}kr}[krj(kr)]^*\cdot\frac{\mathrm{d}}{\mathrm{d}kr}[krh(kr)] + l(l+1)\frac{j(kr)}{kr}^*\frac{h(kr)}{kr}\right), \tag{S21}$$

$$\int_0^{2\pi}\int_0^{\pi} \vec{N}^3_{l,m}(k,\vec{r})\cdot\vec{N}^{1*}_{l,m}(k,\vec{r})\sin\theta \mathrm{d}\theta\mathrm{d}\varphi = \pi j(kr)^*h(kr). \tag{S22}$$

Spatial averaging of the optical chirality enhancement

We use the definition of OCE (f) from the main text (eq. 1) and substitute the fields with their decomposition into incident and scattered fields,

$$f^T = -Z\mathrm{Im}\left((\vec{E}_{scat}+\vec{E}_{inc})^*\cdot(\vec{H}_{scat}+\vec{H}_{inc})\right). \tag{S23}$$

After evaluating the scalar product, the result is

$$f^T = -Z\mathrm{Im}\left(\vec{E}^*_{scat}\cdot\vec{H}_{scat} + \vec{E}^*_{inc}\cdot\vec{H}_{scat} + \vec{E}^*_{scat}\cdot\vec{H}_{inc} + \vec{E}^*_{inc}\cdot\vec{H}_{inc}\right). \tag{S24}$$

We assign a symbol to each term,

$$f^T = f^T_{scat} + f^T_{int,I} + f^T_{int,S} + 1. \tag{S25}$$

Orthogonality of VSHs results in canceling out of any terms in which $m \neq m'$ or $l \neq l'$ and that include products of $\vec{M}$ and $\vec{N}$ or their complex conjugates. We assume that the structure is axially symmetric and hence, its T-matrix is diagonal with respect to m. The left-handed CPL illumination contains only $m = 1$. Thus, from this point we drop index m and assume that it is always $m = 1$. Therefore,

$$f^T_{scat,l} = \frac{1}{4\pi}\int_0^{2\pi}\int_0^{\pi} {b^{mag}}^*_l b^{el}_l \vec{N}_l\cdot\vec{N}_l + {b^{el}}^*_l b^{mag}_l \vec{M}_l\cdot\vec{M}_l \sin\theta \mathrm{d}\theta\mathrm{d}\varphi, \tag{S26}$$

$$f^T_{int,I,,l} = \frac{1}{4\pi}\int_0^{2\pi}\int_0^{\pi} {a^{mag}}^*_l b^{el}_l \vec{N}_l\cdot\vec{N}_l + {a^{el}}^*_l b^{mag}_l \vec{M}_l\cdot\vec{M}_l \sin\theta \mathrm{d}\theta\mathrm{d}\varphi, \tag{S27}$$

$$f^T_{int,S,,l} = \frac{1}{4\pi}\int_0^{2\pi}\int_0^{\pi} {b^{mag}}^*_l e^{el}_l \vec{N}_l\cdot\vec{N}_l + {b^{el}}^*_l a^{mag}_l \vec{M}_l\cdot\vec{M}_l \sin\theta \mathrm{d}\theta\mathrm{d}\varphi. \tag{S28}$$

The integrals are evaluated using Equation S19-Equation S22,

$$
\begin{aligned}
f^T_{scat} &= \frac{1}{4}\sum_l (b^{mag}_l)^* b^{el}_l |h_l(kr)|^2 + \\
&+ \left(\frac{1}{k^2r^2} \left| \frac{\mathrm{d}}{\mathrm{d}kr}[krh_l(kr)] \right|^2 + l(l+1) \left| \frac{h_l(kr)}{kr} \right|^2 \right) (b^{el}_l)^* b^{mag}_l, && \text{(S29)} \\
f^T_{int,I} &= \frac{1}{4}\sum_l j_l(kr)^* h_l(kr) (a^{mag}_l)^* b^{el}_l + \\
&+ \left(\frac{1}{k^2r^2} \frac{\mathrm{d}}{\mathrm{d}kr}[krj_l(kr)]^* \cdot \frac{\mathrm{d}}{\mathrm{d}kr}[krh_l(kr)] + l(l+1) \frac{j_l(kr)}{kr}^* \frac{h_l(kr)}{kr} \right) (a^{el}_l)^* b^{mag}_l, && \text{(S30)} \\
f^T_{int,S} &= \frac{1}{4}\sum_l h_l(kr)^* j(kr) (b^{mag}_l)^* a^{el}_l + \\
&+ \left(\frac{1}{k^2r^2} \frac{\mathrm{d}}{\mathrm{d}kr}[krh_l(kr)]^* \cdot \frac{\mathrm{d}}{\mathrm{d}kr}[krj_l(kr)] + l(l+1) \frac{h_l(kr)^*}{kr} \frac{j_l(kr)}{kr} \right) (b^{el}_l)^* a^{mag}_l. && \text{(S31)}
\end{aligned}
$$

We simplify these equations by inserting the VSWF expansion coefficients for the incident field ($a^{mag}_l = a^{el}_l = \sqrt{2(2l+1)} i^{l-1}$) and use helicity preserving multipoles (eq. 11 in the main text) which leads to the eqs. 13 and 15 in the main text.

Orientation averaging of surface averaged chirality enhancement

We split the calculation of the orientation averaged chirality enhancement into two steps. First, we find the interference terms ($f^T_{int,S}$,$f^T_{int,I}$) and then, we find the scattered field contribution (f^T_{scat}). Each of these terms requires a different orientation averaging procedure.

Calculation of the interference terms requires finding the orientation averaged value of the scattered fields ($\vec{E}_{scat}$,$\vec{H}_{scat}$). We show the procedure for the electric field only as the procedure for the magnetic field is the same. To that end, we insert the T-matrix ansatz (Equation S15) into Equation S10

$$
\begin{aligned}
\vec{E}_{scat} = \sum_{l,m,l',m'} & (T^{mm}_{l,m,l',m'} a^{mag}_{l',m'} + T^{me}_{l,m,l',m'} a^{el}_{l',m'}) \vec{M}_{l,m} + \\
& + (T^{em}_{l,m,l',m'} a^{mag}_{l',m'} + T^{ee}_{l,m,l',m'} a^{el}_{l',m'}) \vec{N}_{l,m}. \qquad \text{(S32)}
\end{aligned}
$$

Note that here we introduce m again not to miss any possible dependence on it. Next, we perform orientation averaging by replacing the T-matrix with its orientation averaged counterpart defined as

$$
\langle T^{i,j}_{l,m,l',m'} \rangle = \delta_{mm'} \delta_{ll'} t^{ij}_l \qquad \text{(S33)}
$$

with

$$
t^{ij}_l = \frac{1}{2l+1} \sum_{m'} T^{ij}_{l,m,l',m'}. \qquad \text{(S34)}
$$

Finally, we realize that the incident field contains only $m = 1$ and perform the summation over m,

$$
\vec{E}_{scat} = \sum_{l,m} \langle b^{mag}_{l,m} \rangle \vec{M}_{l,m} + \langle b^{el}_{l,m} \rangle \vec{N}_{l,m}, \qquad \text{(S35)}
$$

where we introduced $\langle \vec{b} \rangle = \langle T \rangle \vec{a}$. After multiplying by the incident magnetic field, the result is an analogue of Equation S30 an it undergoes the same transformations leading to eq. 17 in the main text.

Finding f^T_{scat} is more involved as it requires finding the orientation average including $b^{mag*} b^{el}$. To that end, we construct a vector of scattered field coefficients $\vec{b} = \begin{bmatrix} b^{mag} \\ b^{el} \end{bmatrix}$ and a vector of incident field coefficients $\vec{a} = \begin{bmatrix} a^{mag} \\ a^{el} \end{bmatrix}$. Then,

$$
f^T_{scat} = \vec{a}^\dagger T^\dagger F T \vec{a}, \qquad \text{(S36)}
$$

where

$$
F = \begin{bmatrix} 0 & |h(kr)|^2 \\ |\frac{\mathrm{d}krh(kr)}{\mathrm{d}kr}|^2 + l(l+1)|\frac{h(kr)}{kr}|^2 & 0 \end{bmatrix}. \qquad \text{(S37)}
$$

Now, because we are finding the product of scattered fields one cannot immediately perform orientation averaging by replacing T with $\langle T \rangle$. Instead we replace the T-matrix with the rotated T-matrix,

$$\tilde{T} = R_{-} T R_{+} \tag{S38}$$

with R_{-} and R_{+} being defined via eq. 1.115 by Doicu et al. [2] After algebraic manipulation, using the fact that F is block diagonal

$$f^{T}_{scat} = \vec{a}^{\dagger} R_{-} T^{\dagger} F T R_{+} \vec{a}. \tag{S39}$$

We find the orientation average of $W = T^{\dagger} F T$, using the procedure outlined by Doicu et al. [2] (see the paragraph leading to eq. 1.123),

$$\langle (W)^{i,j}_{l,m,l',m'} \rangle = \delta_{mm'} \delta_{ll'} \hat{t}^{ij}_{l} \tag{S40}$$

with

$$\hat{t}^{ij}_{l} = \frac{1}{2l+1} \sum_{m'} (W)^{ij}_{l,m,l',m'}. \tag{S41}$$

Then,

$$\langle f^{T}_{scat} \rangle = \vec{a}^{\dagger} \langle W \rangle \vec{a}. \tag{S42}$$

References

[1] Andrey B. Evlyukhin, Carsten Reinhardt, Andreas Seidel, Boris S. Luk'yanchuk, and Boris N. Chichkov. Optical response features of Si-nanoparticle arrays. *Phys. Rev. B*, 82:045404, 2010.

[2] Adrian Doicu, Thomas Wriedt, and Yuri Eremin. *Light scattering by systems of particles: Null-field method with discrete sources: Theory and programs.* Springer-Verlag, 2006.

Supplementary Figures

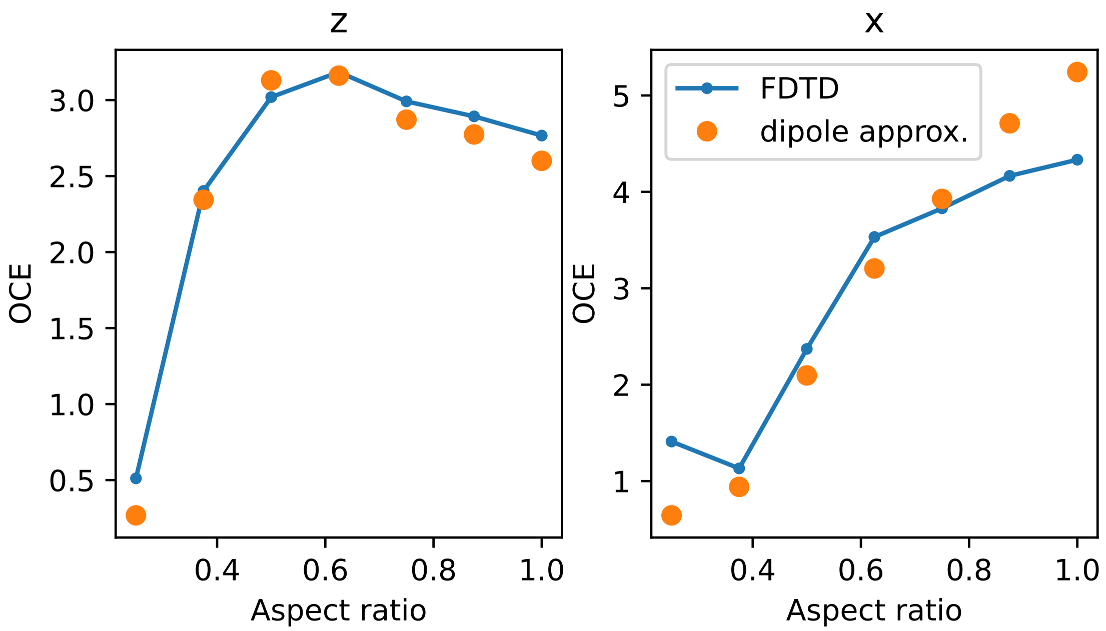

Figure S1: Accuracy of OCE from FDTD and T-matrix. Comparison of OCE along (left) negative z and radial direction for FDTD and T-matrix. The wavelength is adjusted to obtain maximal OCE in each FDTD simulation.

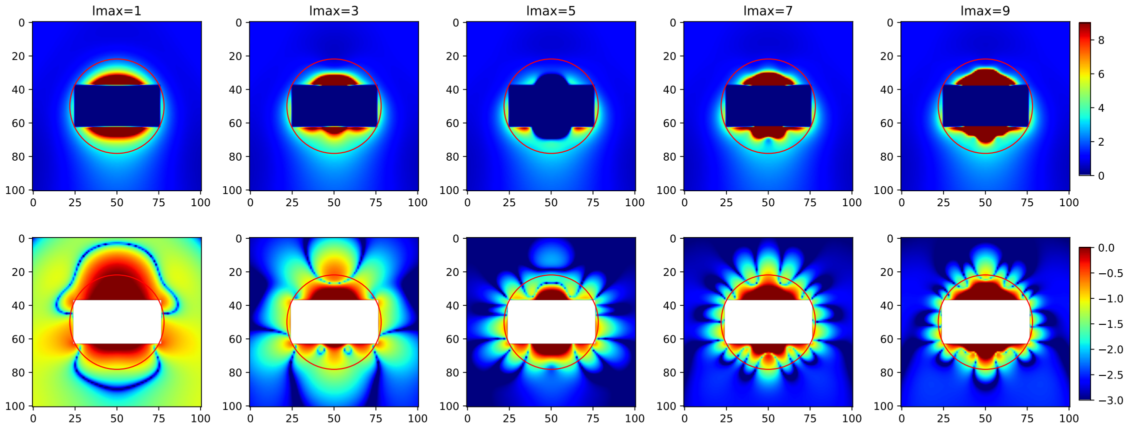

Figure S2: Impact of multipole truncation order on OCE in T-matrix. Top row: OCE, bottom row: FDTD vs T-matrix error. Each column corresponds to a different value of multipole truncation order. The red circles indicate the minimum circumscribing sphere outside which the T-matrix error of the electric fields is greatly reduced in comparison to inside the sphere.

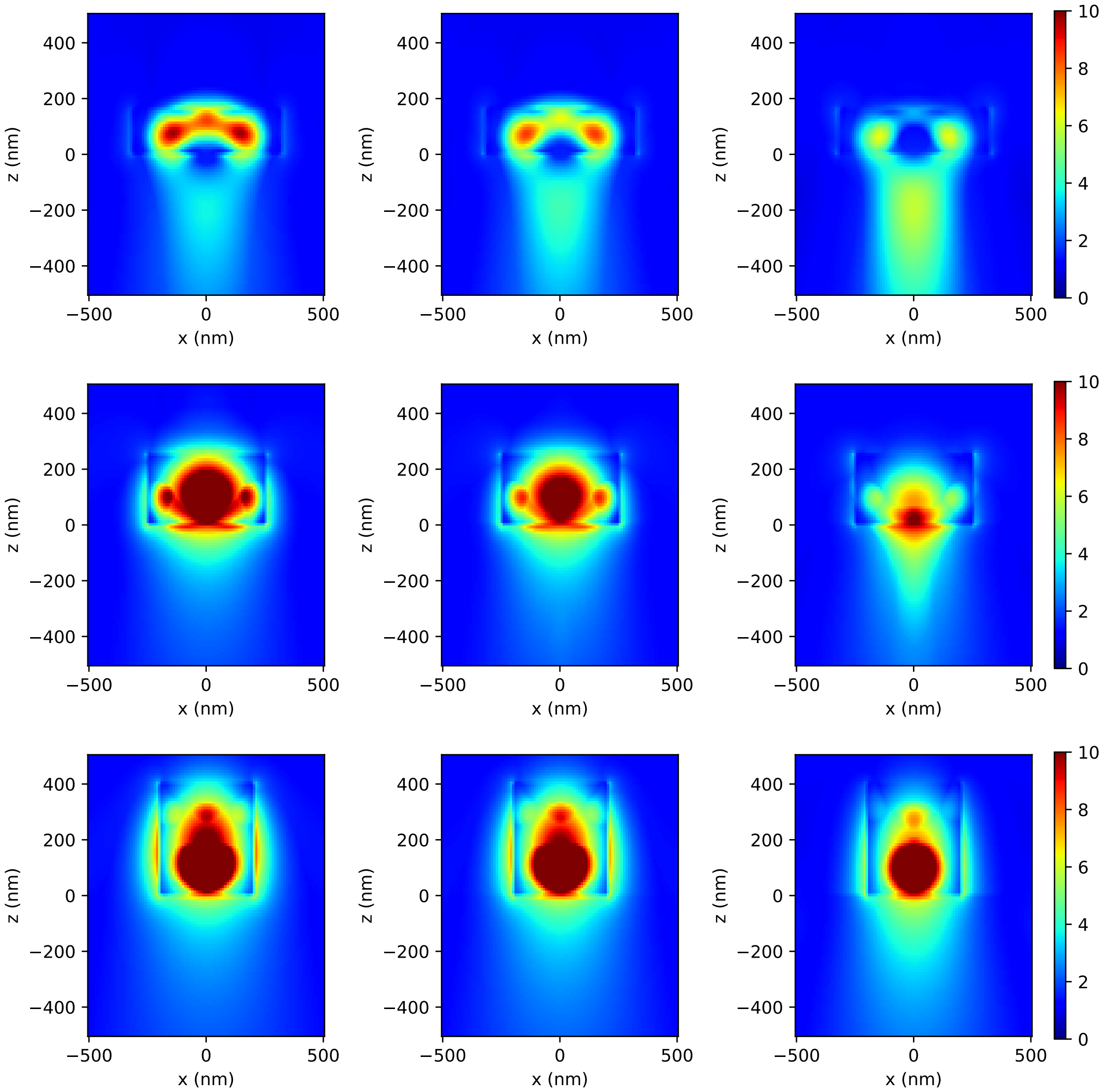

Figure S3: Optical chirality enhancement maps in reflection. Spatial OCE maps obtained with FDTD simulations in reflection configuration (see Figure 6 in the main text). Columns correspond to various n_{sub} values: (left) 1.33, (middle) 1.5, (right) 2.0. Rows correspond to different ARs: (top) 0.25, (middle) 0.5, (bottom) 1.0. The wavelength is adjusted to obtain maximal OCE. y coordinate is fixed at 0.

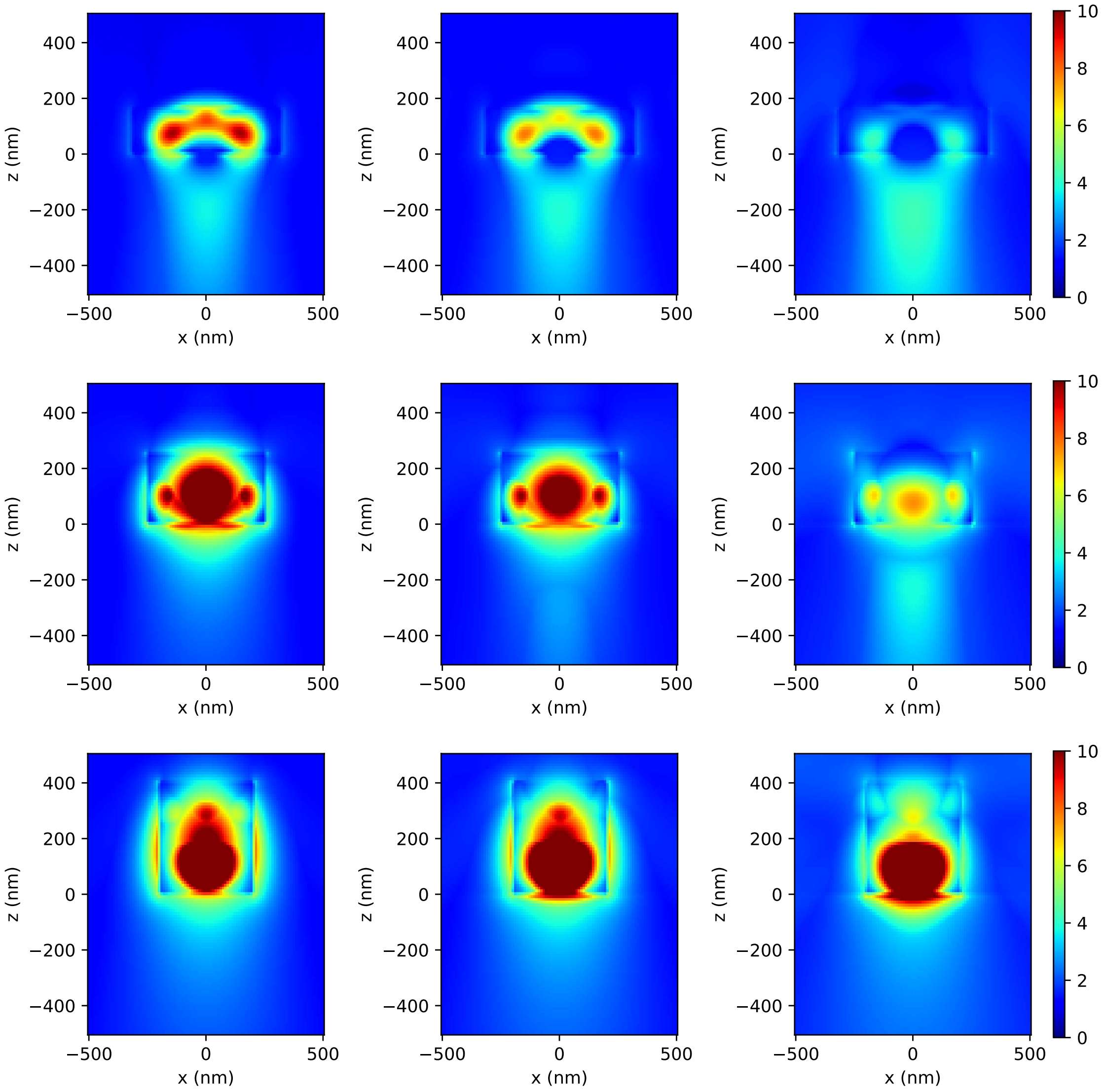

Figure S4: Optical chirality enhancement maps in transmission. Spatial OCE maps obtained with FDTD simulations in transmission configuration (see Figure 6 in the main text). Columns correspond to various n_{sub} values: (left) 1.33, (middle) 1.5, (right) 2.0. Rows correspond to different ARs: (top) 0.25, (middle) 0.5, (bottom) 1.0. The wavelength is adjusted to obtain maximal OCE. y coordinate is fixed at 0.
